# Contralaterally EMG-triggered functional electrical stimulation during serious gaming for upper limb stroke rehabilitation: a feasibility study

**DOI:** 10.3389/fnbot.2023.1168322

**Published:** 2023-05-25

**Authors:** Chiara Höhler, Laura Wild, Alexandra de Crignis, Klaus Jahn, Carmen Krewer

**Affiliations:** ^1^Faculty of Sport and Health Science, Chair of Human Movement Science, Technical University Munich, Munich, Germany; ^2^Department of Neurology, Research Group, Schoen Clinic Bad Aibling, Bad Aibling, Germany; ^3^Ludwig-Maximilians University of Munich (LMU), German Center for Vertigo and Balance Disorders (DSGZ), Munich, Germany

**Keywords:** virtual reality, neuromuscular stimulation, patient treatment, electromyography, hand recovery

## Abstract

**Introduction:**

Virtual Reality/serious games (SG) and functional electrical stimulation (FES) therapies are used in upper limb stroke rehabilitation. A combination of both approaches seems to be beneficial for therapy success. The feasibility of a combination of SG and contralaterally EMG-triggered FES (SG+FES) was investigated as well as the characteristics of responders to such a therapy.

**Materials and methods:**

In a randomized crossover trial, patients performed two gaming conditions: SG alone and SG+FES. Feasibility of the therapy system was assessed using the Intrinsic Motivation Inventory (IMI), the Nasa Task Load Index, and the System Usability Scale (SUS). Gaming parameters, fatigue level and a technical documentation was implemented for further information.

**Results:**

In total, 18 patients after stroke (62.1 ± 14.1 years) with a unilateral paresis of the upper limb (MRC ≤4) were analyzed in this study. Both conditions were perceived as feasible. Comparing the IMI scores between conditions, perceived competence was significantly increased (*z* = −2.88, *p* = 0.004) and pressure/tension during training (*z* = −2.13, *p* = 0.034) was decreased during SG+FES. Furthermore, the task load was rated significantly lower for the SG+FES condition (*z* = −3.14, *p* = 0.002), especially the physical demand (*z* = −3.08, *p* = 0.002), while the performance was rated better (*z* = −2.59, *p* = 0.010). Responses to the SUS and the perceived level of fatigue did not differ between conditions (SUS: *z* = −0.79, *p* = 0.431; fatigue: *z* = 1.57, *p* = 0.115). For patients with mild to moderate impairments (MRC 3–4) the combined therapy provided no or little gaming benefit. The additional use of contralaterally controlled FES (ccFES), however, enabled severely impaired patients (MRC 0–1) to play the SG.

**Discussion:**

The combination of SG with ccFES is feasible and well-accepted among patients after stroke. It seems that the additional use of ccFES may be more beneficial for severely impaired patients as it enables the execution of the serious game. These findings provide valuable implications for the development of rehabilitation systems by combining different therapeutic interventions to increase patients' benefit and proposes system modifications for home use.

**Clinical trial registration:**

https://drks.de/search/en, DRKS00025761.

## 1. Introduction

Due to demographic change, stroke is becoming more prevalent (Feigin et al., 2021). Worldwide, there are more than 10 million new cases each year, and more than 100 million people suffer from stroke sequalae. Stroke is the second leading cause of death and a major cause of disability (Feigin et al., 2021). Up to 40% of survivors have long-term limitations in activities of daily living (ADLs) and often rely on caregivers or are institutionalized (Luengo-Fernandez et al., 2013). Therefore, stroke is of immense public health relevance because of the burden it places on family members, the health care system, and society (Crichton et al., 2016).

A stroke often results in sudden onset of neurological symptoms like hemiparesis and hemihypesthesia, speech and visual problems, balance disturbances, and neuropsychological symptoms such as aphasia, apraxia, agnosia, and neglect. Symptoms depend on the brain area affected and usually involve a combination of several impairments. Survival probabilities after an ischemic stroke improved over the past decades (Rücker et al., 2020). Improvements in stroke management and treatment may have contributed to this. However, 15-years after stroke about one-third of survivors was living with a mild disability and one-third with a moderate or severe disability. The latter suffer long-term impairments in basic ADLs, such as dressing, and in performing instrumental ADLs such as preparing meals (Desrosiers et al., 2003; Crichton et al., 2016). Therefore, stroke survivors are often dependent on caregivers or are institutionalized (Luengo-Fernandez et al., 2013). Upper limb function is fundamental to ADLs and important for independence. Recovery of arm function is targeted by various rehabilitative intervention strategies with the overall goal of being less dependent in daily living (Desrosiers et al., 2003; Pollock et al., 2014; Platz, 2021). These intervention strategies are based on underlying mechanisms of neuroplasticity and principles of motor learning (Meier, 2021). Therefore, training has been shown to be effective for motor recovery when it is repetitive (Veerbeek et al., 2014; French et al., 2016), intensive (Pollock et al., 2014; Platz, 2021), task specific (Kleim and Jones, 2008; Veerbeek et al., 2014) and variable (Veerbeek et al., 2014; French et al., 2016). Moreover, feedback and motivation are also important for learning to be effective. The success of traditional therapies is limited and current rehabilitation methods often do not adequately incorporate evidence based on motor learning theories (Maier et al., 2019a). Therefore, new approaches are needed to address these problems. Both, functional electrical stimulation (FES) and Virtual Reality (VR)/serious gaming (SG) therapies are used in stroke rehabilitation (Pollock et al., 2014; Platz et al., 2020). Increasingly, the use of VR technologies in therapeutic interventions for neurorehabilitation is also being discussed and researched. VR technologies provide a multisensory environment that promotes brain neuroplasticity and thus contributes to the rehabilitation of motor disorders (Teo et al., 2016). Often, VR technological interventions incorporate elements of gamification to make therapy interesting and motivating (Doumas et al., 2021). Such games, which are used for education and rehabilitation purposes, are referred to as serious games (SG) (Doumas et al., 2021). Those are specifically designed to facilitate brain plasticity and recovery by incorporating principles of motor learning (Maier et al., 2019b) and provide the user with task-specific and repetitive training, which can be individualized to the patient's ability and motivates the user (Saposnik and Levin, 2011; Lohse et al., 2014; Veerbeek et al., 2014; Laver et al., 2017; Maier et al., 2019a). A Cochrane review, and a consecutive review published in 2021 could confirm a positive effect on motor recovery when VR technologies were used as an adjunct to conventional therapy (Laver et al., 2017; Bui et al., 2021). To enable intensive training, even in severely affected individuals with hemiplegia, electrostimulation seems to be an appropriate therapeutic method (Oujamaa et al., 2009; Meadmore et al., 2012). Electrical stimulation can be used in a functional context, referred to as FES, to assist impaired or absent function during a task (Moe and Post, 1962; Doucet et al., 2012). FES applications can be orthotic applications aiming at replacing a function or therapeutic applications which target the regain of a function. While the orthotic application on the upper limb has not been studied a lot, the positive effects of therapeutic FES interventions include improvements in muscle strength (Veerbeek et al., 2014; Küçükdeveci et al., 2018), motor function (de Kroon et al., 2005; Veerbeek et al., 2014; Hebert et al., 2016; Küçükdeveci et al., 2018; Monte-Silva et al., 2019), range of motion (Veerbeek et al., 2014), and ADLs (Veerbeek et al., 2014; Howlett et al., 2015; Eraifej et al., 2017). In addition, it has been shown that involving the patient's voluntary effort with EMG-triggered FES is more effective compared to passive stimulation (de Kroon et al., 2005). A combination of both approaches seems promising and has been investigated in only a few studies so far (e.g., Meadmore et al., 2012; Buick et al., 2016; Kumar et al., 2016; Collaborators GBDLRoS et al., 2018; Lee et al., 2018; Fu et al., 2019; Chou et al., 2020; Norouzi-Gheidari et al., 2021). In all of these studies task-specific training via VR-based games was provided that incorporated FES. Various VR and gaming devices (e.g., smart glove gaming system, touch table screen, computer screens), as well as different FES systems (e.g., custom-built FES wristlet, single electrodes, and electrode arrays) were used for this purpose. Additionally, two studies also integrated an arm support system, such as SaeboMAS (Kutlu et al., 2016) or an unweighting exoskeleton robotic system (Meadmore et al., 2012). All games were designed to promote upper limb motor recovery including reaching, grasping and object manipulation tasks, which often mimicked ADLs such as opening a door, pressing a button, or positioning an object.

A combined approach may have the potential to positively impact treatment outcomes, as FES and SG complement each other in terms of motor learning principles that are important for effective neurorehabilitation interventions (Fu et al., 2019). In addition, the combined use of VR/SG and FES provides multimodal feedback (visual, auditory, and proprioceptive), which may further enhance the therapeutic effect (Lee et al., 2018). Preliminary results have shown improvements in motor function (Meadmore et al., 2012; Buick et al., 2016; Kumar et al., 2016; Kutlu et al., 2016; Lee et al., 2018; Fu et al., 2019; Norouzi-Gheidari et al., 2021), range of motion (Kutlu et al., 2016), and cognitive function (Fu et al., 2019). The combined interventions were also found to be interesting, motivating and challenging (Buick et al., 2016; Fu et al., 2019), and to reduce the burden on clinical therapists (Chou et al., 2020). However, there is limited evidence to support this novel approach, and most studies included small numbers of patients, had no control group and/or examined therapies for home use in chronic stroke patients. Moreover, controlling FES stimulation still appears to be a challenge (Meadmore et al., 2012; Buick et al., 2016; Kutlu et al., 2016; Lee et al., 2018). Existing control mechanisms were either inaccurate, did not require voluntary patient effort, or were complex, expensive and time-consuming and therefore not feasible in the hospital setting. In contrast, using a contralaterally controlled FES with kinematic sensing gloves seems feasible for home use (Fu et al., 2019). A similar approach using FES with the contralateral unimpaired hand controlled via EMG with a commercially available stimulation device also appears feasible and easy to use but has not yet been studied in combination with SG (Krewer et al., 2008). Accordingly, the aim of the present work is to investigate whether the combination of SG and contralaterally EMG-triggered FES is feasible, and what factors might influence the feasibility of the combined therapy system. Another objective is to investigate the benefit from the additional use of FES while playing SG.

## 2. Materials and methods

### 2.1. Participants

Patients in an inpatient rehabilitation hospital (Schoen Clinic Bad Aibling) were screened for study eligibility based on the following inclusion criteria: (i) ischemic or hemorrhagic stroke, (ii) age ≥18 years, (iii) cognitively able to follow instructions, (iv) no pain or low pain level in wrist or fingers of both limbs (Numerical Rating Scale/Pain scale <4), (v) functional impairments in wrist and fingers of one limb (Medical Research Council scale score (MRC) ≤4), (vi) no rigid spasticity in the affected limb (Modified Ashworth Scale (MAS) ≤3), and (vii) able to sit in a chair for the duration of the session (about 1 h). Study-related and device-related exclusion criteria were (i) pregnancy, (ii) severe psychiatric disorders, (iii) active implantable devices (e.g., pacemaker), or other metal implants within the stimulation area, (iv) severe or frequent epileptic seizures in the past, (v) cancer, and (vi) wounds in the application area of the electrodes or measuring equipment. In addition, patients with no sensitivity in wrist or fingers and no motion resulting from FES (e.g., due to atrophy or polyneuropathy) were excluded from the study. A botulinum toxin injection during study participation led to study termination. The study was approved by the Ethics Committee of the Ludwig-Maximilians University (LMU) Munich, Germany (registration number: 21-0270), and registered with the German Clinical Trials Register (registration number: DRKS00025761).

### 2.2. Study design

**Figure 1:**
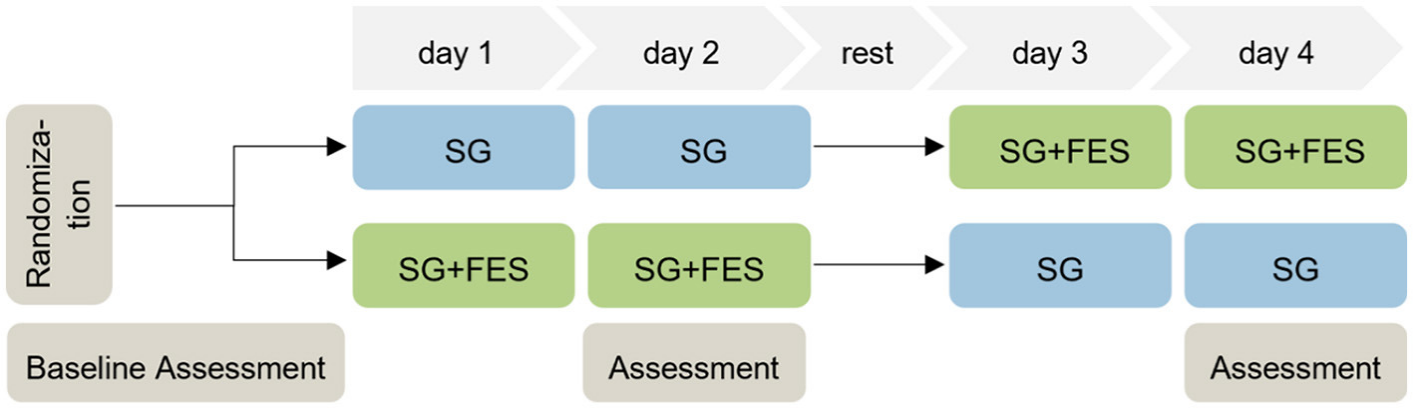
Scheme of the study design.

In this randomized crossover trial, the feasibility of serious gaming (SG) was compared to SG supported by FES (SG+FES). Each participant first underwent a baseline assessment to determine their current functional status. Patients received two consecutive training sessions of SG alone (control condition) and two consecutive training sessions of SG+FES (experimental condition). Whether they started with or without FES support, was randomized with an allocation ratio of 1:1. Randomization with different block sizes of four and six, was done using sealed envelopes. Due to the nature of the trial, patients, therapists and assessors were not blind to the group allocation. Each session lasted about 45 to 60 min. Between the conditions at least an 1-day washout period was scheduled to lower the risk of carryover effects from the previous intervention (Dwan et al., 2019). All four sessions were completed within the timeframe of 2 weeks. The schematic of the study design is illustrated in Figure 1.

### 2.3. Intervention

The intervention in each condition consisted of performing serious gaming exercises with (SG+FES) and without (SG) FES support of the affected hand. Study participation did not affect the standard therapies during the rehabilitation stay, which took place to the usual extent.

**Figure 2:**
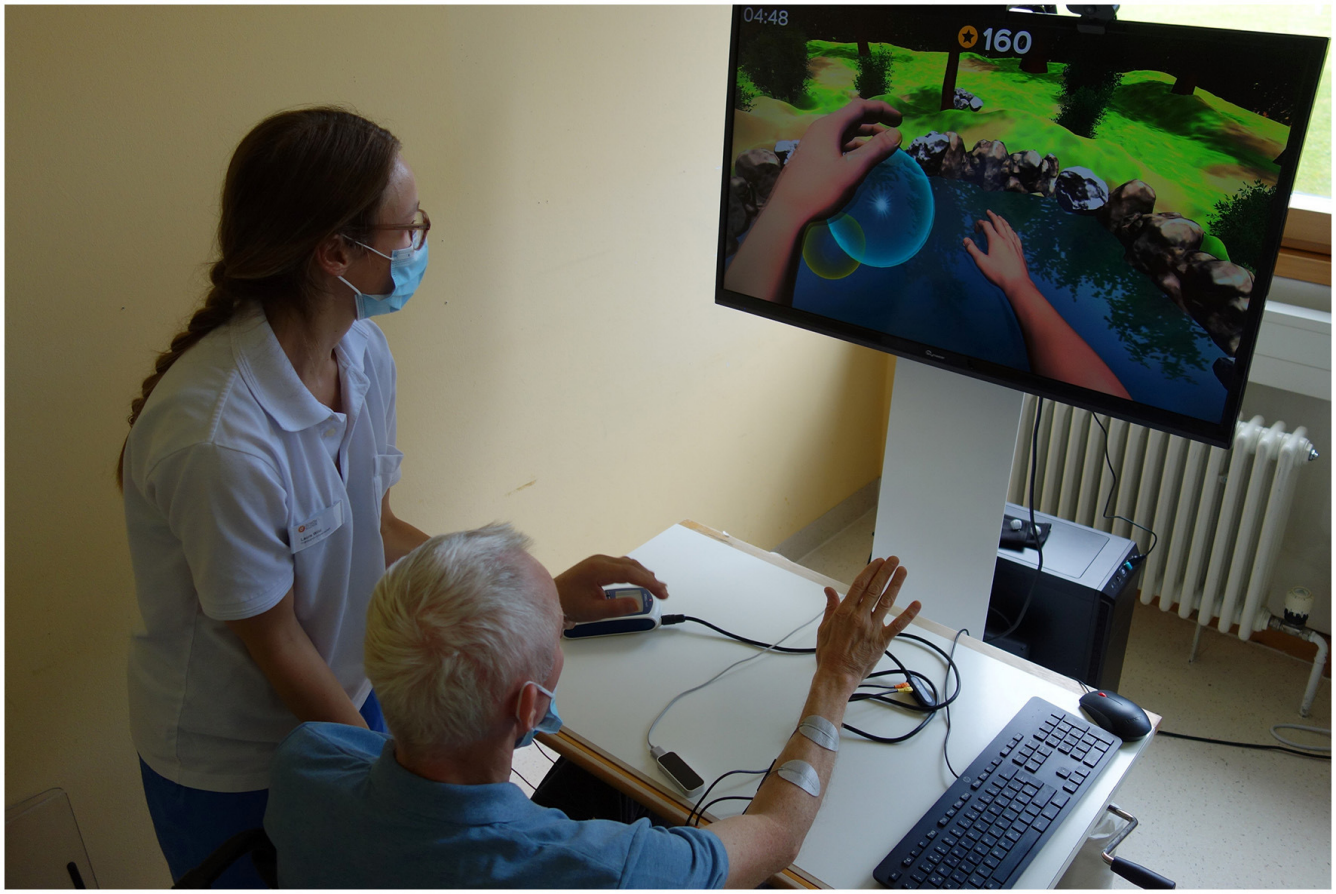
The Bubbles scenario (SG+FES) of the Rehabilitation Gaming System, including a motion sensor in front of the patient to track wrist and finger movements as well as a mouse and keyboard to change gaming parameters. A therapist is providing proximal support. Stimulating electrodes are placed on the left wrist extensors, EMG electrodes on the right wrist extensors. The patient's informed consent was obtained for publishing photo material.

For the gaming scenario, the Rehabilitation Gaming System (RGS; Eodyne Systems S.L., Barcelona, Spain), a VR-based rehabilitation tool, was used in both conditions. The RGS platform provides gamified and engaging exercises for effective and functional recovery of motor and cognitive functions, validated in stroke patients (Cameirão et al., 2009, 2011; Ballester et al., 2017). The system integrates a motion sensor (Leap Motion, Inc., San Francisco, United States) to capture hand movements in real time, which can be mapped to a hand- and forearm-like virtual avatar on a computer screen, allowing the player to interact with the game scenario. In the current trial, the *Bubbles* scenario was used, which targets grasping, reaching and bi-manual coordination. In this scenario, bubbles rise from a lake which need to be burst to score points by catching the bubbles with an open hand, and then closing the hand to bust. The exercise can be tailored to the user. The side of appearance of the bubbles can be adjusted. For this trial, the side of appearance of bubbles was set to the patient's paretic side to encourage the use of the impaired hand. Furthermore, the size of the bubbles can be set according to the patient's ability to open and close the hand, and the speed of the game can be adjusted by increasing the frequency of the rising bubbles. Size and speed were adjusted for each patient individually. Regarding the size, the smaller the bubbles, the less the user must open the hand, but the more they must close it to make them burst. Accordingly, smaller bubbles were chosen for patients who could not open their hand completely, or who had good ability in closing the hand, to make the task challenging. As the size equaled different scoring values, ranging from very large (one point per bubble) to very small (ten points per bubble), size and speed were adjusted only at the beginning of each game and documented for each patient to make the sessions comparable. Patients were seated in front of a large screen on which the game was displayed. The motion sensor was placed on the table in front of the patient. If needed, the proximal part of the arm (i.e., the elbow and forearm) was supported and guided by the therapist to move the arm toward the bubble, allowing the patient to concentrate on opening and closing the hand. The current total score was constantly visible at the top of the screen, and in the end of each game the performance was ranked in comparison with previous games. A briefing and explanation of the game scenario was scheduled for the first session. For each session a total game duration of 30 min was targeted, which was divided into three separate games of 10 min each. For patients who were unable to play the game due to insufficient hand opening to grasp a bubble, the session was terminated after a few minutes. Otherwise, there was a short break between the games to reduce muscle fatigue. The set-up of the system is shown in Figure 2.

**Figure 3:**
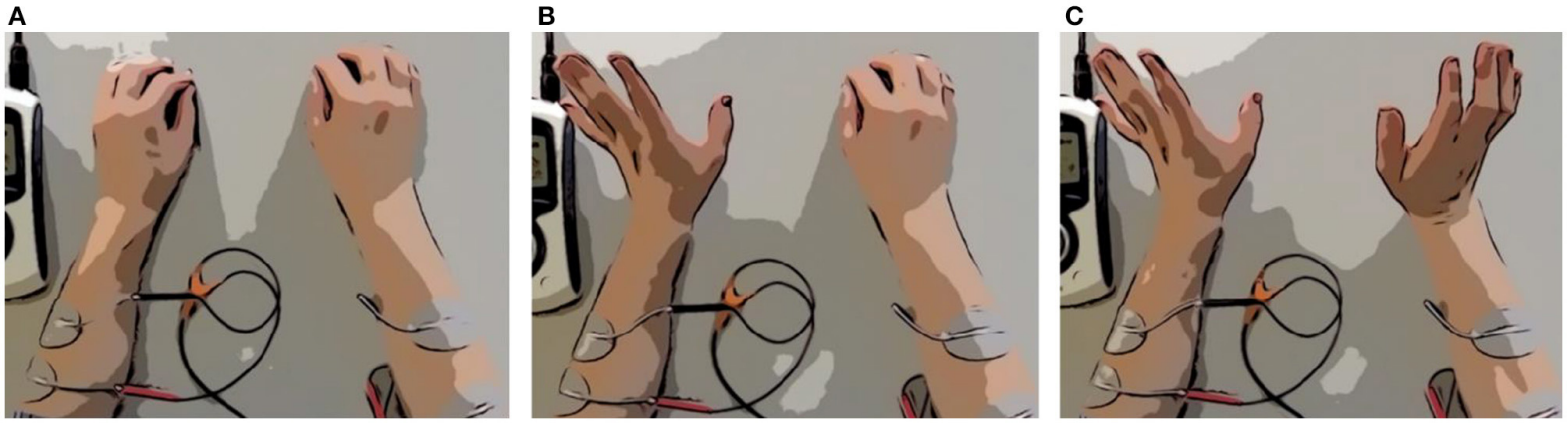
Scheme of EMG-based ccFES. Two electrodes are placed on each forearm. First, both hands are closed **(A)**. Then the non-paretic hand (left hand) opens **(B)**. Muscle activity is recorded by EMG and exceeds a set threshold. Stimulation of the affected paretic hand (right hand) is triggered. Wrist and finger extensor muscles of the affected hand contract and the hand opens **(C)**.

During the SG+FES session, finger and wrist extension were supported by electrical stimulation using the STIWELL med 4 (MED-EL Elektromedizinische Geräte GmbH, Innsbruck, Austria). To facilitate finger and wrist extension, a pair of self-adhesive electrodes was attached to each forearm targeting extensor carpi ulnaris, extensor digitorum communis, and partially also extensor carpi radialis muscles (Krewer et al., 2008). The myoelectric activity from the non-paretic contralateral side was used to trigger the stimulation when a set EMG threshold was reached. Further, we refer to this form of stimulation as contralaterally controlled FES (ccFES). Thus, patients could decide at which time the stimulation should be triggered. The opening of the affected hand is then delayed by about one second after activating the hand extensor muscles of the non-paretic limb. The schematic of EMG-based ccFES is illustrated in Figure 3.

Biphasic square-wave pulses with a frequency of 35 Hz and a pulse width of 250 μs were used for stimulation. The muscle contraction/relaxation time ratio was set to 2/1 s on/off time. Therefore, 2 s of stimulation were followed by one second of pause. The stimulation included no ramping-up and ramping-down period to insure fast reaction on the game. However, in patients with instable wrists, a ramping-up and ramping down of 0.5 s was added to reduce strain on the structures. The stimulation intensity (mA) and EMG-threshold (μV) was set individually for each patient. Therefore, the intensity was slowly increased until the first muscle twitch was seen and was further increased to the level that produced maximum wrist and finger extension without discomfort or pain. The EMG threshold was set to trigger stimulation when the non-paretic hand was opened without much effort. For integrating the stimulation to the gaming therapy, there are two different ways: Either the patient opens the unaffected hand first, which triggers the stimulation and leads to the opening of the affected hand, or the patient opens both hands simultaneously and the function of the affected hand is supported by the stimulation. The second option is only possible if the patient can open the hand volitionally. The procedure of the game in the SG+FES condition is as follows: (i) A bubble rises from the lake, (ii) patient opens his unaffected hand/both hands at the same time, (iii) the muscle activity exceeds the EMG threshold and triggers the stimulation on the affected side, (iv) short time later the affected hand opens/the hand opening improves, (v) the hand motion is detected by the sensor and displayed as virtual motion by the avatar, (vi) the digital avatar catches the bubble with the opened hand, (vii) the bubble attaches to the avatar's hand, (viii) the patient closes the affected hand volitionally (including use of gravity) or with the support of the therapist, (ix) the bubble bursts and points are collected.

### 2.4. Outcome parameters

Baseline patient characteristics including sociodemographic characteristics, severity of impairment, cognition (Montreal Cognitive Assessment, MoCA) (Nasreddine et al., 2005), pain, and technical affinity were collected before the interventions. For more details see Table 1. Assessments were performed immediately after completing each condition (see Figure 1).

**Table 1:** Demographic and clinical patient characteristics (*n* = 18).

**Parameter**	***M (SD), min-max*; or number**
Age [years]	62.1 (14.1), 40–86
Sex (men/women)	15/3
Type of stroke (ischemia/hemorrhage)	11/7
Side of paresis (left/right)	7/11
Time after stroke [months]	2.4 (1.6), 1.0–7.3
**Fugl-Meyer Assessment (FMA)**	
FMA—upper extremity motor function (0–66)	26.1 (18.6), 4–59
FMA—hand/part C (0–14)	6.4 (4.6), 0–13
FMA—sensory function (0–12)	9.1 (3.7), 0–12
Categorization (severe, 0–28/moderate, 29–42/mild, 43–66)	11/3/4
Montreal Cognitive Assessment (MoCA, 0–30)	23.7 (5.6), 10–28 (12 normal, 2 mild, 4 moderate)
Modified ashworth scale (max value; 0/1/1+/2/3)^*^	5/4/5/2/2
Tardieu scale (max value; 0/1/2/3/4)^*^	5/2/7/4/0
**Medical research council scale** ^*^	
Finger extensors (0/1/2/3/4/5)	3/8/1/2/4/0
Wrist extensors (0/1/2/3/4/5)	4/6/1/4/3/0
**Mean maximum grip strength [kg]**	
Paretic hand	4.9 (6.4), 0–22.8
Non-paretic hand	30.5 (11.0), 7.6–48
Technical Affinity questionnaire—Total score (TA-EG, 0–5)	3.6 (0.7), 2.5–4.5
TA-EG—Enthusiasm (0–5)	3.6 (1.1), 1.6–5.0
TA-EG—Competence (0–5)	3.8 (0.9), 1.8–5.0
TA-EG—Positive attitude (0–5)	3.9 (0.8), 2–5
TA-EG—Negative attitude (0–5)	3.2 (0.6), 1.8–4.4

A MoCA score of 26 or above is considered normal; a score from 18–25 is considered a mild, 10–17 a moderate, and less than 10 points a severe cognitive impairment.

* individual values are shown in Supplemental Table 1.

#### 2.4.1. Primary outcomes

Feasibility of the therapy system was assessed after each condition using the Intrinsic Motivation Inventory (IMI), the Nasa Task Load Index (NASA-TLX), the System Usability Scale (SUS), and the vertical numerical rating scale (NRS-FRS) for perceived fatigue. Patients either filled the questionnaires by themselves or with the support of the supervising researcher.

The patients' motivation during the respective interventions was assessed using the IMI, a multidimensional questionnaire designed to evaluate motivational structures for performing given tasks in laboratory experiments (McAuley et al., 1989; Dec et al., 1994). In recent years, the use of the IMI has become widespread in stroke rehabilitation research. Monardo, Pavese (Monardo et al., 2021) also recommend the use of the IMI to assess patient motivation and satisfaction during technology-assisted rehabilitation. For the purpose of the study, a shorter version with a total of 20 items was selected (Bergmann et al., 2018). It contains the following five subscales relevant for our study: interest/enjoyment, value/usefulness, effort, perceived competence, and felt pressure and tension. Four items per subscale were included and rated on a seven-point Likert scale from “strongly disagree”/1 to “strongly agree”/7. The original questionnaire was translated into German and slightly modified to adapt them to the given task. The subscale *interest/enjoyment* directly reflects the patient's intrinsic motivation, whereas the other concepts influence intrinsic motivation and self-regulatory behavior. For example, it is assumed that individuals internalize and become self-regulating when they can identify with an activity's value (Dec et al., 1994). The total score and the score per subscale were calculated by averaging the respective items.

The NASA-TLX is a multidimensional rating scale for measuring a person's subjectively perceived workload during or shortly after completing a given task (Hart and Staveland, 1988). The measurement tool comprises a total of six subdimensions of workload: mental demand, physical demand, temporal demand, performance, effort, and frustration. The subscales are presented on straight lines with the endpoints low and high on which patients mark the point that best represents their subjective perception of workload of the given task (NASA Ames Research Center, 1986). For scoring, the line is divided into 20 equal intervals marked by vertical ticks. The position of the marker is then rated numerically on a scale from 0 to 100, with five points for each interval. If a subject marks between two ticks, the value is rounded up. The value of each subscale is measured and represents a unique score. The mean value of the subscales gives the overall RAW-TLX score between 0 and 100, whereas a higher score indicates greater perceived workload (Hart, 2006). The German version of the RAW-TLX was used. The term NASA-TLX is retained in the following for simplification.

Testing usability during and after the development of a product or system is an incredibly important process (Peres et al., 2013). The patients' perceived usability of the therapy system (SG and SG+FES) was assessed using the SUS. Thereby usability is defined as the appropriateness of a system or tool to a purpose or to a context in terms of effectiveness (success), efficiency (effort), and satisfaction (level of comfort). The SUS comprises a total of ten items, which are rated on a five-point Likert scale from “strongly disagree”/0 to “strongly agree”/4 (Brooke, 1996). The SUS was proven to be a valid and reliable instrument (even with small samples) for assessing the overall perceived usability of a wide range of products and services (including health care devices). In this study, a translated German version of the SUS was used and the term “system” was changed to “therapy system” for better suitability (Bangor et al., 2008). In addition, a rating scale was used to determine the degree to which the patient perceived ccFES as supportive or as disturbing. The rating scale consists of a ten-centimeter horizontal line divided into 20 equal intervals marked by vertical ticks with the endpoints “disturbing”/0 and “supportive”/10. The higher the value, the more supportive the ccFES was perceived to be.

After each therapy session, the level of perceived fatigue was assessed using a vertical numerical rating scale supplemented by faces (NRS-FRS) (Chuang et al., 2015). The tool is easy to administer and has shown a high sensitivity and specificity in assessing fatigue intensity in patients with stroke. In addition, compared to the normal numerical rating scale (NRS), it may be more suitable for patients who lack cognitive and visuospatial functions. The NRS-FRS scale consists of a ten-centimeter vertical line with a rating scale from “no fatigue”/0 to “worst possible fatigue”/10 and six facial expressions (from smiling to crying). Patients were asked to rate their perceived overall fatigue level by pointing to a number on the scale that best represented it. Based on the score assigned, fatigue can be categorized as no fatigue (0), mild (1–3), moderate (4–6), or severe fatigue (7–10).

Lastly, adverse events were documented.

#### 2.4.2. Secondary outcomes

Gaming parameters (duration and score) were recorded during each session to assess the orthotic efficacy of the therapy system. The gaming duration was defined as the amount of time spent performing the serious game per session, whereas the score was defined as the total number of points achieved during therapy per session. To make the scores comparable between the sessions and conditions, the total number of points were divided by the level of difficulty (bubble size). Thus, the number of points also corresponds to the number of successful hand opening and closing repetitions. The patient's performance was automatically saved in a remote medical information management system and additionally noted on a documentation sheet. For both parameters, the mean value for each condition was used for analyses.

### 2.5. Data and statistical analysis

Descriptive statistics (mean, M; standard deviation, SD; median, Mdn; Q1–Q3, quartile 1–quartile 3) are used to describe the study population and outcome variables. Outcome variables were first tested for normality using the Shapiro-Wilk test. For between-condition comparisons, paired-sample *t*-tests or Wilcoxon signed-rank tests were performed. In addition, subgroup Wilcoxon tests were performed with classification of patients based on disease severity and spearman rank correlations (non-parametric test for ordinal or metric data) were used to analyze relationships between feasibility and (1) time since stroke, (2) age, (3) technical affinity, and (4) degree of cognitive impairment. The alpha level was set to.05. Inferential analysis was performed using IBM SPSS Statistics 27 and visualization was done in RStudio.

## 3. Results

### 3.1. Baseline

**Figure 4:**
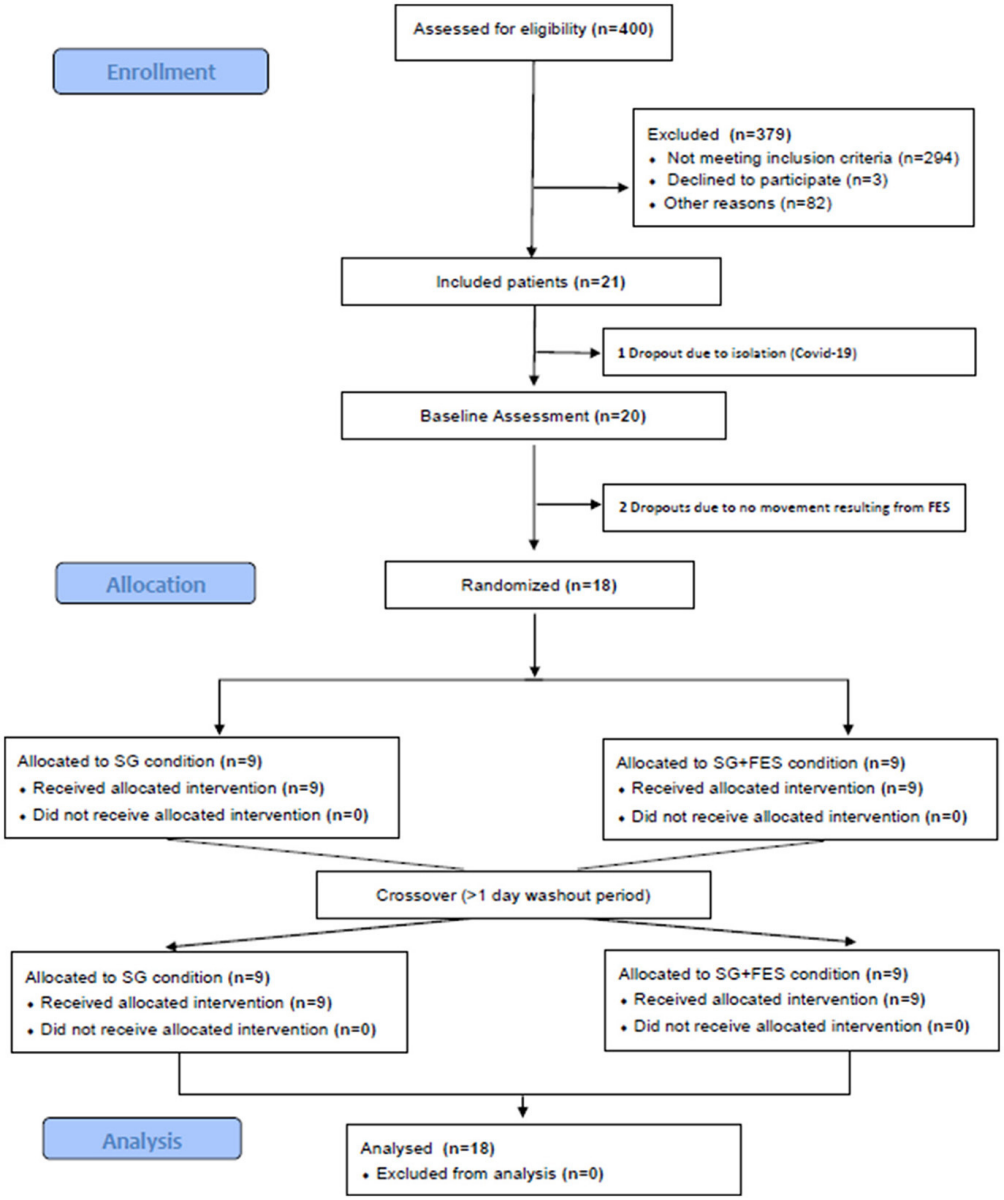
The patients' flow through the study. SG, Serious Gaming; SG+FES, contralaterally EMG-triggered FES.

Between June and September 2021, and April and November 2022 inpatients at the Schoen Clinic Bad Aibling were screened for eligibility (see criteria in Section 2.1). Figure 4 shows the patients' flow through the study. Of 400 patients who were assessed for eligibility, 294 patients did not meet the previously defined inclusion criteria. The top three reasons for not meeting the inclusion criteria were: only fine motor impairments in the paretic hand (inclusion criteria v), not being able to sit upright for more than 1 h (inclusion criteria vii), or not being able to follow instructions due to cognitive or language impairments (inclusion criteria iii), in 21%, 13%, or 12% of the excluded patients, respectively. Three patients declined to participate. Other reasons for not being able to participate (*n* = 82) were e.g., a hospital stay shorter than 2 weeks, or a language barrier due to the inability to understand or speak German.

In the end, 21 of the screened patients were included in the study of which one patient dropped out before study participation and two patients after baseline assessment (see Figure 4). Demographic and clinical characteristics of analyzed patients (*n* = 18) are shown in Table 1.

### 3.2. Primary outcome parameters

The IMI, NASA-TLX and SUS questionnaires as well as the perceived fatigue level provide subjective measures of feasibility. Questionnaire results are reported for both conditions in Table 2.

**Table 2:** Feasibility scores for both conditions and the inferential comparison between conditions.

**Outcome parameters**	**SG+FES *Mdn (Q1–Q3)***	**SG *Mdn (Q1–Q3)***	**Statistical values**
IMI (0–7)^*^	5.5 (5.3–5.8)	5.3 (4.8–5.5)	*z* = −1.66, *p* = 0.097
Interest/enjoyment	7.0 (6.8–7.0)	6.7 (6.2–7.0)	*z* = −1.11, *p* = 0.265
Value/usefulness	6.8 (6.0–7.0)	6.8 (6.2–7.0)	*z* = −0.61, *p* = 0.539
Effort	7.0 (6.4–7.0)	6.8 (5.5–7.0)	*z* = −0.72, *p* = 0.473
Perceived competence	5.9 (4.8–6.6)	4.9 (2.5–5.5)	*z* = −2.88, *p* = 0.004
Pressure/tension	1.8 (1.0–2.5)	2.3 (1.0–3.0)	*z* = −2.13, *p* = 0.034
NASA-TLX (0–100)^*^	32.9 (22.9–46.9)	49.2 (37.1–58.7)	*z* = −3.14, *p* = 0.002
Mental demand	47.5 (25.0–76.3)	50.0 (17.5–53.8)	*z* = −0.42, *p* = 0.678
Physical demand	50.0 (13.8–76.3)	72.5 (50.0–90.0)	*z* = −3.08, *p* = 0.002
Temporal demand	42.5 (18.8–50.0)	50.0 (23.8–50.0)	*z* = −0.54, *p* = 0.593
Performance	20.0 (10.0–37.5)	52.5 (25.0–96.3)	*z* = −2.59, *p* = 0.010
Effort	30.0 (20.0–52.5)	62.5 (23.8–50.0)	*z* = −1.85, *p* = 0.064
Frustration	5.0 (0.0–25.0)	12.5 (3.8–31.3)	*z* = −1.07, *p* = 0.284
SUS (0–100)^*^	85.0 (71.9–90.6)	82.5 (74.4–88.1)	*z* = −0.79, *p* = 0.431
Perceived fatigue level (0–10)^*^	4.5 (2.3–6.5)	2.5 (0.0–6.1)	*z* = 1.57, *p* = 0.115

IMI, Intrinsic Motivation Inventory; NASA-TLX, Nasa Task Load Index; SG, Serious Gaming; SG+FES, Serious Gaming plus contralaterally EMG-triggered FES; SUS, System Usability Scale.

* individual values are shown in Supplementary Table 2.

Comparing the IMI scores between conditions, the statistical values indicate a significant increase in the patients' perceived competence (*z* = −2.88, *p* = 0.004) and a decrease in the perceived pressure/tension during ccFES supported training (*z* = −2.13, *p* = 0.034). Furthermore, the task load was rated significantly lower in the SG+FES condition (*z* = −3.14, *p* = 0.002). The subscale of physical demand showed lower values (*z* = −3.08, *p* = 0.002) and performance higher values (*z* = −2.59, *p* = 0.010) for the SG+FES condition. Responses to the SUS did not differ significantly between conditions (*z* = −0.79, *p* = 0.431). Also, the perceived level of fatigue showed no significant difference (*z* = 1.57, *p* = 0.115).

The technical and user documentation revealed that the addition of ccFES was perceived as very supportive (*Mdn* = 9.0, *Q1–Q3* = 7.8–10.0). However, 16 of the 18 patients (mild: 2/4, moderate: 3/3, severe: 11/11) needed proximal support of the paretic arm, which was given by the therapist.

### 3.3. Factors influencing feasibility

A subgroup analysis reveals the severity of the hemiparesis as a factor that had an influence on the feasibility of using SG+FES compared to SG alone. Mildly and moderately impaired patients perceived no difference in feasibility between conditions (*p* ≥ 0.068). However, patients with a severe hemiparesis remarked a significantly higher task load during the therapy when they were not supported by ccFES (*Mdn*_SG_ = 50.0, *Q*1−−*Q*3_SG_ = 41.7–58.3, *Mdn*_SG+FES_ = 35.8, Q1–Q3_FES+SG_ = 16.7–47.5, *z* = −2.1, *p* = 0.033). Furthermore, their intrinsic motivation was higher when playing the game with ccFES support (*Mdn*_SG_ = 4.9, *Q*1−−*Q*3_SG_ = 4.6–5.5, *Mdn*_SG+FES_ = 5.5, *Q*1−−*Q*3_SG+FES_ = 5.3–5.8, *z* = −2.0, *p* = 0.046).

The patients' technical affinity showed a positive correlation (*r* = 0.46) with the feasibility of the SG+FES condition, assessed by the SUS, which is tending toward statistical significance (*p* = 0.053). Factors such as age, time since stroke and the degree of cognitive impairment (according to the MoCA) did not correlate with any feasibility assessment of the SG+FES condition (*p* ≥ 0.192).

### 3.4. Secondary outcome parameters

**Figure 5:**
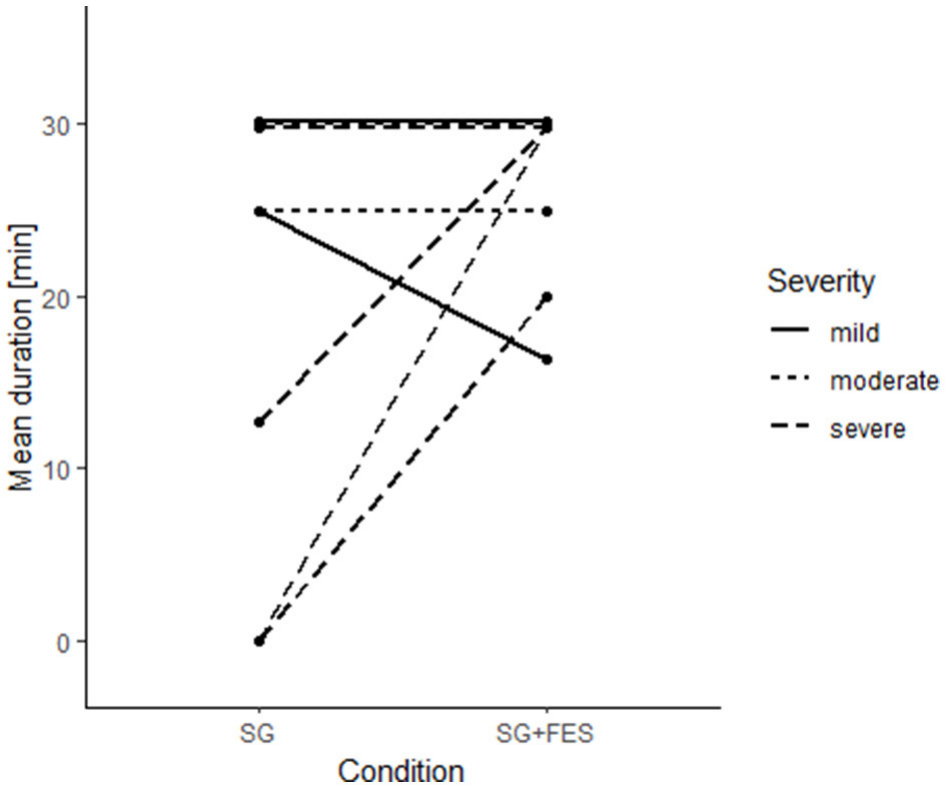
Individual changes (*n* = 17) in therapy duration [min].

Since one patient (severely impaired) received physical support in hand opening and closing by the therapist, the online effect of ccFES was analyzed in 17 patients. Gaming parameters (i.e., the gaming score and the session duration) were compared between SG+FES and SG condition. Overall, the support by ccFES showed a significant effect on the gaming duration (*z* = −2.41, *p* = 0.016) enabling longer training times. With the support of ccFES, patients played on average 27.7 (*SD* = 4.5, *min* = 16.3) minutes, while the training lasted on average 17.8 (*SD* = 14.2, *min* = 0) minutes without ccFES. In Figure 5, the difference in gaming duration is grouped according to the severity of impairment. For patients with a mild or moderate hemiparesis, the therapy duration was not expanded by the addition of ccFES support. However, only few severely impaired patients (4/10), were able to play the therapy game without ccFES support at all (10.3 ± 14.2 min), but the support by ccFES enabled them to train on average 28.0 (*SD* = 4.2) minutes (*z* = −2.41, *p* = 0.016). Overall, the gaming duration of ccFES supported trials ranged from 20 to 30 min. Factors preventing the patients from finishing the 30 min of therapy included muscle fatigue, general fatigue, shoulder pain, and time pressure due to the patient's therapy schedule.

**Figure 6:**
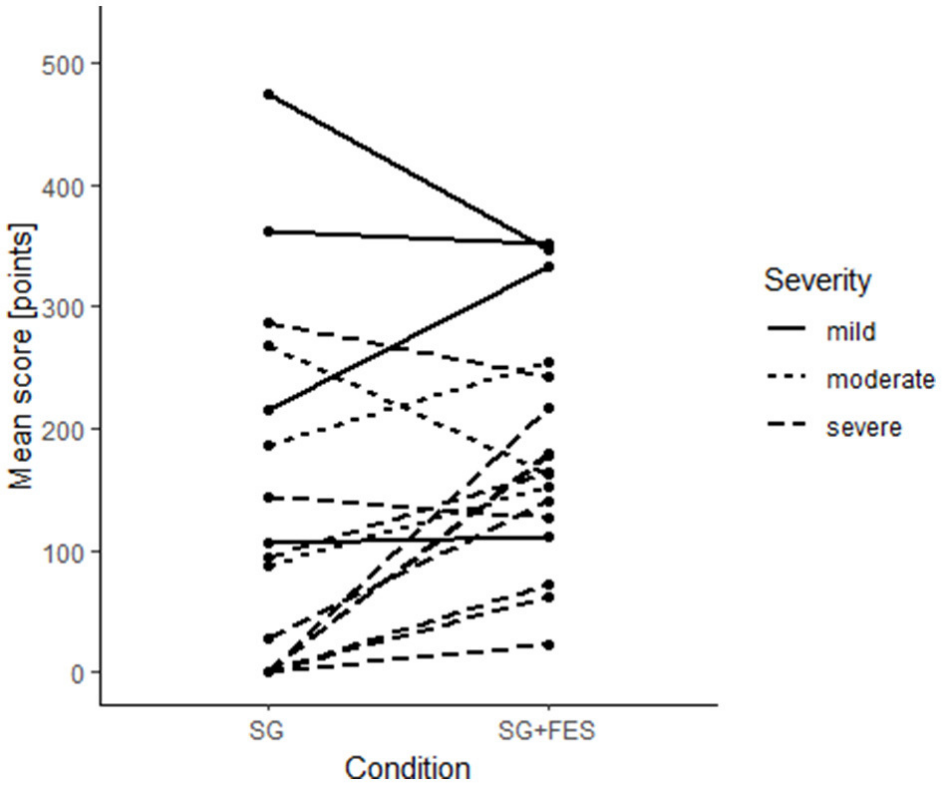
Individual changes (*n* = 17) in gaming score [points].

With respect to the gaming score, patients achieved a 50.5 points higher score when they were supported by ccFES (183.0 ± 97.5 points) compared to no support (132.5 ± 146.2 points). This effect reached statistical significance (*t*_(16)_ = −2.17, *p* = 0.045). Subgroup analyses reveal a significant online effect of ccFES leading to an increase of on average 84.9 (*95%CI* = 22.8–147.0) points in the group of severely impaired patients (*t*_(9)_ = −3.09, *p* = 0.013). As visualized in Figure 6, there is no clear pattern for mildly and moderately impaired patients; some profited from ccFES support, others achieved less points in the SG+FES condition.

### 3.5. Harms

Neither FES nor the gaming scenario itself led to any serious adverse events. By following the manufacturer's instructions, device-related exclusion criteria were set to reduce the risk of harms. During recruitment, attention was given to not include patients with allergies to adhesive material. Reactions to the stimulation include temporary redness of the skin, most likely induced by an increase in blood flow. Pain in the shoulder or at the back of the hand was recorded as side effect of the general intervention by two patients, leading to an earlier termination of the therapy session (after 15–20 min).

## 4. Discussion

### 4.1. Feasibility of the system

The feasibility of combining a SG application with ccFES was investigated within the population of subacute stroke patients in the design of a randomized crossover study. It was demonstrated that the combination of SG with EMG-based ccFES is feasible, safe and well-accepted among patients after stroke. Overall, both conditions were perceived as motivating, and were rated to be at an appropriate task load. The SUS score was high in both conditions, with 85.0 points in SG and 82.5 points in SG+FES condition, which is considered as good according to the existing literature (Bangor et al., 2008, 2009). Thus, the results indicate that both systems are easy to learn for most people, easy to use without much technical support and not too complex, inconsistent or awkward. In addition, most subjects felt confident in using the therapy systems and could imagine using those regularly. The results of the IMI show that both conditions were with a median score of 5.5 (SG+FES) and 5.3 (SG) perceived as enjoyable and valuable, and thus patients were motivated during therapy. This is in line with previous work (Buick et al., 2016; Fu et al., 2019; Doumas et al., 2021). When ccFES support was provided, patients perceived a significantly higher level of competence and experienced significantly less pressure. This is also evident in the results of the NASA-TLX, which show a significantly lower workload when the movement was support by ccFES with a median of 32.9 points, compared so SG only with a median of 49.3 points. Specifically, the physical and temporal demand was significantly lowered by ccFES. In general, patients rated the stimulation as supportive rather than distracting. However, almost 90% of patients needed proximal anti-gravity support in order to participate in the therapy. This points out the strong need of adding a proximal robotic component (e.g., lightweight exoskeleton) to the system.

Our results thus proof that the combination of SG and EMG-based ccFES is as feasible as SG alone, and in some aspects even superior to SG alone. According to previous research, the implementation of such a combined therapy is expected to have additional benefits on motor recovery. By adding ccFES to SG, the following principles are incorporated and complement each other. SG delivers individualized, task-specific training in a multisensory environment, including visual and auditory feedback on performance and results (Cameirão et al., 2009). In addition, it promotes the active execution of movements of the paretic limb and observation of movement through an avatar representation on the computer screen (Cameirão et al., 2009). Moreover, controlling FES with the contralateral side encourages bilateral arm movements, which can be beneficial in improving motor recovery after stroke (Cauraugh and Kim, 2002). Doumas, Everard (Doumas et al., 2021) demonstrated that a greater focus on these principles increases the efficacy of SG therapies.

The investigation of factors that influence the feasibility of the combined system revealed the severity of the upper limb hemiparesis as one aspect. Especially, severely impaired patients rated the SG+FES condition as motivating and supportive, in terms of reducing the task load. Since the age of the participants, the time since stroke and the degree of cognitive impairments had no influence on the feasibility of the combined training, the results imply no restrictions in therapy prescription. However, it has to be highlighted that the time since stroke for the investigated study cohort was seven months or shorter (without specification in the inclusion and exclusion criteria). Solely, technical affinity is a potential influencing factor of feasibility. Patients with a higher technical affinity tend to rate the combined approach even more positive, potentially because they are in general more open for new technologies and less afraid to use them.

### 4.2. Orthotic effect

Only patients with sufficient residual movement activity were able to play the game under the SG condition without ccFES, while it was possible for all patients under the SG+FES condition regardless of impairment. This becomes evident in the significant increase in therapy time in the combined training compared to unassisted SG. More than half of the severely impaired patients were not able to perform the game without assistance, so ccFES enabled them to execute the SG at all. Obviously, this group of severely impaired patients were those who had the strongest orthotic effect in terms of therapy duration. ccFES facilitated these patients to increase the therapy intensity and the number of repetitions of hand opening, which could potentially lead to an increased rehabilitation outcome for the severely affected patients.

When comparing the success achieved in the game indicated by the resulting scores, ccFES led to an orthotic effect. However, only the performance of patients with a severe hemiparesis significantly improved under ccFES support. In contrast, patients with mild to moderate impairment showed little or no gain in their performance in the SG+FES condition.

Although moderately and mildly impaired patients did not show any orthotic effect, a therapeutic effect of the combined SG+FES application might still be possible due to the increased sensory information. It is worth to highlight that the control of the additional ccFES component did not lead to any disadvantage, neither in feasibility nor in work load. A therapeutic effect, however, needs to be investigated in future studies, as this was not addressed in the here presented trial. That would even mean to focus on different outcome parameters to investigate the therapeutic effect (e.g., Action Research Arm Test, Barthel Index, Functional Independence Measure).

### 4.3. Strengths and limitations

To our knowledge, this is one of the few studies demonstrating the feasibility of combining serious gaming with ccFES using a randomized controlled design and the first study using ccFES for this purpose. Some study-related weaknesses, however, were identified that limit the interpretability and generalizability of the results. The patients and the research team were not blinded with respect to the applied interventions. However, the research team itself did not rate feasibility nor the orthotic efficacy of the system. Rather was the assessment of the orthotic effect captured by means of the gaming system. Also, regarding the subjective report of feasibility by the participating patients, the patients have to be aware of the different gaming conditions to better evaluate the feasibility. Therefore, the potential risk of bias due to the lack of blinding is considered to be low. Despite possible biases due to self-reporting, such as recall bias, the inclusion of patients' opinions and needs in a participatory design is necessary for the rehabilitation system to be user-friendly and accepted by patients. Since the vast majority of patients did not show cognitive impairments, it is not expected that many patients could not recall the therapy sessions, which were performed right before answering the questionnaires. Regarding the sample size, it is important to note that the focus of this work was to demonstrate the feasibility of the concepts of combining serious gaming with EMG-based ccFES in a hospital setting, instead of testing the efficacy in upper limb rehabilitation. Future work is needed to verify these results with a larger number of participants, especially to perform higher powered sub-group analysis according to the severity of the hemiparesis. Lastly, there are some limitations of the system when thinking about using it at home. For an application of the system at home, it is necessary to allow the patient to train without support of a therapist, and therefore the following implications were derived. The goal of the *Bubbles* scenario of the RGS system was to burst bubbles by opening and closing the hand. The extension of the fingers and the opening of the hand were supported by ccFES. However, to successfully score points, finger bending is also essential, which was not supported in the current study. For patients with very limited flexion, it was not possible to play the game without the therapist manually closing the hand. Therefore, not only the extensor but also the flexor muscles of the fingers should be stimulated with ccFES. Furthermore, the bubble game of the RGS system required a good proximal arm function to be able to hold the hand independently over the motion sensor and thus to be able to play the game. However, since proximal arm function was not sufficient in almost all patients and no arm support system was planned for the study, the therapist had to physically assist the proximal joints

Our findings provide valuable implications for the development of rehabilitation systems by combining different therapeutic interventions to increase patients benefit. The combined approach provides individualized, task-specific rehabilitation with the potential to increase therapy intensity, especially for severely impaired patients with muscle weakness, and to maintain patients' motivation and engagement. To bring this therapy system from a hospital setting to home use, the integration of an additional arm support system (e.g., exoskeleton) would be necessary, as shown to be feasible in two studies investigating SG+FES (Meadmore et al., 2012; Kutlu et al., 2016). Such a hybrid combination of robotic and FES has already been studied and makes a proportion of about 25% of existing hybrid systems, as shown in a recent review (Höhler et al., 2023). As another solution, either in addition or as an alternative to FES, the distal functions could also be supported by integrating a hand support system [e.g., hand exoskeleton (Prange-Lasonder et al., 2017)]. The combined use of robotic and FES support for hand functions, however, has not yet been studied (Höhler et al., 2023).

## Data availability statement

The original contributions presented in the study are included in the article/Supplementary material, further inquiries can be directed to the corresponding author.

## Ethics statement

The studies involving human participants were reviewed and approved by Ethics Committee of the Ludwig-Maximilians University (LMU) Munich, Germany. The patients/participants provided their written informed consent to participate in this study.

## Author contributions

Conceptualization: AC and CK. Methodology: CH, AC, and CK. Formal analysis and visualization: CH and LW. Investigation: LW, AC, and KJ. Resources and supervision: KJ and CK. Data curation: LW and AC. Writing—original draft: CH, LW, and CK. Writing—review and editing: AC and KJ. Project administration: AC, KJ, and CK. Funding acquisition: CK. All authors contributed to the article and approved the submitted version.
