# Supplementary material for: Contralaterally EMG-triggered functional electrical stimulation during serious gaming for upper limb stroke rehabilitation: a feasibility study
Source: Front Neurorobot. 2023 May 25;17:1168322. doi: 10.3389/fnbot.2023.1168322 (PMC10248145; doi:10.3389/fnbot.2023.1168322)
Supplement: Supplementary file 1 [file Table_1.docx]

Supplementary Material

Contralaterally EMG-triggered Functional Electrical Stimulation during Serious Gaming for Upper Limb Stroke Rehabilitation: a Feasibility Study

**Supplementary Table 1.** Individual values of baseline data.

|  | ***Modified Ashworth Scale*** | | | | | | ***Tardieu Scale*** | | | | | | ***Medical Research Council Scale*** | | | | | |
| --- | --- | --- | --- | --- | --- | --- | --- | --- | --- | --- | --- | --- | --- | --- | --- | --- | --- | --- |
|  | *elbow* | | *wrist* | | *finger* | | *elbow* | | *wrist* | | *finger* | | *elbow* | | *wrist* | | *finger* | |
|  | *fl.* | *ex.* | *fl.* | *ex.* | *fl.* | *ex.* | *fl.* | *ex.* | *fl.* | *ex.* | *fl.* | *ex.* | *fl.* | *ex.* | *fl.* | *ex.* | *fl.* | *ex.* |
| 1 | 1 | 1 | 1 | 0 | 2 | 0 | 2 | 1 | 1 | 0 | 1 | 0 | 2 | 2 | 1 | 1 | 1 | 1 |
| 2 | 1.5 | 1.5 | 0 | 0 | 0 | 0 | 1 | 2 | 0 | 0 | 0 | 0 | 4 | 4 | 3 | 3 | 3 | 3 |
| 3 | 1.5 | 1 | 1.5 | 1.5 | 1 | 0 | 2 | 1 | 2 | 2 | 1 | 0 | 3 | 4 | 3 | 3 | 3 | 3 |
| 4 | 3 | 0 | 3 | 0 | 2 | 0 | 2 | 0 | 2 | 0 | 1 | 0 | 1 | 1 | 1 | 0 | 1 | 1 |
| 5 | 1.5 | 1.5 | 1 | 1 | 0 | 0 | 2 | 2 | 1 | 1 | 0 | 0 | 5 | 4 | 4 | 4 | 4 | 4 |
| 6 | 0 | 0 | 0 | 0 | 0 | 0 | 0 | 0 | 0 | 0 | 0 | 0 | 0 | 0 | 0 | 0 | 0 | 0 |
| 7 | 1.5 | 3 | 3 | 0 | 2 | 0 | 0 | 2 | 3 | 0 | 1 | 0 | 1 | 1 | 1 | 1 | 1 | 1 |
| 8 | 1.5 | 1 | 1.5 | 0 | 0 | 0 | 2 | 1 | 2 | 0 | 0 | 0 | 1 | 1 | 0 | 0 | 0 | 0 |
| 9 | 1.5 | 1 | 1.5 | 0 | 1.5 | 0 | 2 | 1 | 3 | 0 | 1 | 0 | 2 | 1 | 2 | 1 | 2 | 1 |
| 10 | 1 | 0 | 0 | 0 | 0 | 0 | 2 | 0 | 0 | 0 | 0 | 0 | 3 | 4 | 4 | 3 | 3 | 4 |
| 11 | 0 | 0 | 0 | 0 | 0 | 0 | 0 | 0 | 0 | 0 | 0 | 0 | 2 | 1 | 1 | 1 | 2 | 1 |
| 12 | 0 | 0 | 0 | 0 | 0 | 0 | 0 | 0 | 0 | 0 | 0 | 0 | 5 | 4 | 3 | 4 | 3 | 2 |
| 13 | 1 | 1 | 0 | 0 | 0 | 0 | 1 | 1 | 0 | 0 | 0 | 0 | 2 | 3 | 2 | 2 | 1 | 1 |
| 14 | 0 | 0 | 0 | 0 | 0 | 0 | 0 | 0 | 0 | 0 | 0 | 0 | 1 | 2 | 1 | 0 | 1 | 0 |
| 15 | 0 | 1 | 0 | 0 | 0 | 0 | 0 | 1 | 0 | 0 | 0 | 0 | 4 | 4 | 4 | 4 | 4 | 4 |
| 16 | 0 | 1 | 1 | 0 | 2 | 0 | 0 | 2 | 3 | 0 | 1 | 0 | 2 | 3 | 2 | 1 | 3 | 1 |
| 17 | 1 | 1 | 1 | 0 | 1 | 0 | 2 | 1 | 3 | 0 | 2 | 0 | 2 | 2 | 1 | 1 | 2 | 1 |
| 18 | 0 | 0 | 0 | 0 | 0 | 0 | 0 | 0 | 0 | 0 | 0 | 0 | 3 | 4 | 3 | 3 | 4 | 4 |

**Supplementary Table 2.** Individual data of primary outcome assessments.

|  | ***IMI*** | | ***NASA-TLX*** | | ***SUS*** | | ***Fatigue*** | |
| --- | --- | --- | --- | --- | --- | --- | --- | --- |
|  | *SG+FES* | *SG* | *SG+FES* | *SG* | *SG+FES* | *SG* | *SG+FES* | *SG* |
| 1 | 5.0 | 4.6 | 16.7 | 42.5 | 85.0 | 82.5 | 1.0 | 0.0 |
| 2 | 5.4 | 5.4 | 26.7 | 45.0 | 85.0 | 92.5 | 1.5 | 1.5 |
| 3 | 5.8 | 5.3 | 28.3 | 23.3 | 70.0 | 82.5 | 2.5 | 6.5 |
| 4 | 5.2 | 4.8 | 12.5 | 57.5 | 75.0 | 87.5 | 4.5 | 0.0 |
| 5 | 4.7 | 5.1 | 40.8 | 50.0 | 50.0 | 60.0 | 3.0 | 4.0 |
| 6 | 5.8 | 4.6 | 46.7 | 65.8 | 72.5 | 60.0 | 5.0 | 0.0 |
| 7 | 5.8 | 3.7 | 47.5 | 58.3 | 92.5 | 100.0 | 3.0 | 0.0 |
| 8 | 5.7 | 4.1 | 35.8 | 41.7 | 90.0 | 82.5 | 6.0 | 0.0 |
| 9 | 6.1 | 4.9 | 30.0 | 48.3 | 100.0 | 90.0 | 3.0 | 0.0 |
| 10 | 5.7 | 5.9 | 44.2 | 60.0 | 77.5 | 87.5 | 5.0 | 7.0 |
| 11 | 5.5 | 5.5 | 45.8 | 37.5 | 92.5 | 82.5 | 7.5 | 2.5 |
| 12 | 4.7 | 4.9 | 49.2 | 65.8 | 57.5 | 70.0 | 7.5 | 6.0 |
| 13 | 5.5 | 5.9 | 66.7 | 72.5 | 55.0 | 77.5 | 6.5 | 5.5 |
| 14 | 5.3 | 5.5 | 61.7 | 52.5 | 77.5 | 72.5 | 7.5 | 7.5 |
| 15 | 5.4 | 5.5 | 25.0 | 35.8 | 85.0 | 87.5 | 1.0 | 2.5 |
| 16 | 6.0 | 5.9 | 5.8 | 10.0 | 97.5 | 100.0 | 0.0 | 0.0 |
| 17 | 5.4 | 5.3 | 27.5 | 50.0 | 85.0 | 75.0 | 6.5 | 6.5 |
| 18 | 5.6 | 5.3 | 6.7 | 33.3 | 87.5 | 80.0 | 4.5 | 5.5 |
